# The impact of smartphone use on working memory in college students: a functional near-infrared spectroscopy study

**DOI:** 10.3389/fpsyt.2025.1725048

**Published:** 2026-01-26

**Authors:** Wenyue Cao, Jiaying Hu, Jiaoyan Wang, Feng Lin, Huaide Qiu

**Affiliations:** 1 School of Rehabilitation and Physical Education, Nanjing Normal University of Special Education, Nanjing, Jiangsu, China; 2 Department of Rehabilitation Medicine, The Affiliated Sir Run Run Hospital of Nanjing Medical University, Nanjing, Jiangsu, China; 3 Department of Rehabilitation Medicine, The First Affiliated Hospital of Nanjing Medical University, Nanjing, Jiangsu, China

**Keywords:** smartphone use, working memory, fNIRS, college students, 2-back task

## Abstract

**Background:**

Excessive screen time among college students is increasingly prevalent and may impair executive functions, particularly working memory (WM). However, the behavioral and neural mechanisms remain unclear.

**Methods:**

A total of 42 college students participated in the experiment and were assigned to either a high screen time group (HSTG) or a low screen time group (LSTG). Brain activity was measured with functional near-infrared spectroscopy (fNIRS) covering the frontal, temporal, and parietal regions during the 2-back working memory task. Group differences in behavioral performance (accuracy, reaction time, false alarms), task-related activation, functional connectivity, and graph-theoretical network were analyzed.

**Results:**

LSTG participants demonstrated significantly higher accuracy and hit rates than those in HSTG, while no group differences were observed in reaction time or false alarm rate. Neuroimaging analyses revealed greater activation in bilateral dorsolateral prefrontal cortex (DLPFC-R: p< 0.001, DLPFC-L: p = 0.007) as well as premotor and supplementary motor cortex (PreM & SMC-R, p = 0.007) in LSTG. Functional connectivity was higher in LSTG at whole-brain (p = 0.047), intra-hemispheric (right: p = 0.022, left: p = 0.049), and inter-hemispheric levels (p = 0.033). Graph-theoretical results further indicated lower clustering coefficient (p = 0.040) and network density (p = 0.035) in HSTG, although global and local efficiency did not differ between groups.

**Conclusion:**

High screen exposure is linked to reduced working memory accuracy, weaker prefrontal engagement, and disrupted network organization, which suggests reliance on less efficient neural strategies. Screen use may thus represent a modifiable factor affecting cognition and brain networks.

## 1 Introduction

The past decade has witnessed rapid increase in smartphone use in people’s daily life (1). In China, there are over 1.1 billion smartphone users, representing approximately 99.4% of the population, with the majority being young and middle-aged individuals. On average, individuals now spend approximately 30.6 hours per week online (2). Recent surveys show that university students often engage in prolonged smartphone use, frequently exceeding eight hours per day, with a significant proportion reporting problematic or addictive use patterns (3).

This pervasive use has raised concerns about its potential impact on cognitive functions. A growing body of research has linked excessive smartphone use to deficits in executive functions (EFs), including cognitive flexibility (4), inhibitory control (5–7), and working memory(WM) (8–11). WM is a cognitive system responsible for temporarily storing and manipulating information necessary for complex tasks such as learning, reasoning, and comprehension (12). It relies heavily on the prefrontal cortex (PFC) (13), which continues to mature through early adulthood. WM capacity has been shown to predict academic achievement, problem-solving efficiency, and self-regulation (14). Given the high neural plasticity of the PFC in young adults, external factors such as intensive smartphone use may influence WM elated brain function (9).

Research on the relationship between smartphone use and WM has produced mixed findings, broadly falling into three categories: some studies suggest negative effects, others report no association, and some studies highlight potential benefits. Several studies suggest detrimental effects. For instance, Sharifian et al. (15) reported that frequent social media use may foster long-term dependence on external memory sources, potentially impairing the brain’s memory functions. Similarly, Abramson et al. (16) found that heavier mobile phone use was associated with reduced accuracy in WM and associative learning tasks, as well as faster but less accurate responses in both simple and complex cognitive tasks among over 300 secondary school student. These findings have been interpreted through several theoretical lenses. First, distraction and cognitive load accounts argue that smartphones deplete cognitive resources needed for WM and alter the top-down attentional patterns (6, 17). Second, emotion regulation mechanism indicated that frequent or intensive social media use increases negative affect, which in turn mediates cognitive interference on working memory (18). In contrast, other studies have reported either null or even positive effects. Pablo et al. reports that no relationship between media usage and WM performance were found across six WM experiments with both online and in-person tasks (19). Conversely, certain digital activities have been associated with cognitive enhancement. For example, cognitive training via specific social media applications or video games has been shown to improve memory in older adults (20, 21), and video game training can enhance WM capacity in younger populations (22, 23). More recently, evidence indicates that active screen time may selectively benefit visual WM (8), and fNIRS data (24) suggest that individuals with PSU can exhibit WM advantages extending from network-related to neutral stimuli, associated with altered prefrontal activation and enhanced frontopolar-DLPFC connectivity. Together, these findings support neuroplasticity accounts, which emphasize that the highly malleable prefrontal cortex in young adults may allow certain digital activities to strengthen prefrontal cortex function and have the training effect on WM processes (24).

Despite the growing body of research, there is a lack of consensus on the extent to which smartphone use affects WM in college students, and the underlying neural mechanisms remain underexplored. Functional near-infrared spectroscopy (fNIRS) is a non-invasive optical imaging technique that measures cortical hemodynamic responses associated with neural activity. Its portability and tolerance to movement make it well-suited for cognitive experiments outside of highly constrained laboratory settings (25, 26). In studies of WM, fNIRS has been widely used to monitor prefrontal activation and functional connectivity during tasks such as the N-back paradigm (11, 27). In the present study, fNIRS provides a practical tool to examine how varying levels of smartphone use influence WM performance and underlying brain activity in college students.

To address these gaps, the present study employs a 2-back working memory paradigm to systematically examine how high levels of electronic screen use influence cognitive performance in college students. Specifically, we investigate the effects of screen-use duration on behavioral outcomes, task-evoked cortical activation patterns, functional connectivity, and large-scale network organization using fNIRS. We hypothesize that higher screen-use duration will be associated with poorer working memory performance, altered brain activation during the task, and disruptions in functional connectivity and network topology.

## 2 Materials and methods

### 2.1 Participants

All participants were full-time undergraduate students spanning first to third year students at a normal university in JiangSu province, China, majoring in Education, Psychology, Rehabilitation Science, or Special Education. Participation was voluntary, and students provided written informed consent prior to data collection. Inclusion criteria were: (1) aged 18–25 years, (2) currently enrolled in undergraduate studies, and (3) normal or corrected-to-normal vision. Exclusion criteria included: (1) history of neurological or psychiatric disorders, (2) use of medications affecting cognitive function, or (3) prior participation in neuroimaging studies within the past three months. This study was approved by the Ethics Committee of Nanjing Normal University of Special Education (IRB #20250924021), and informed consents were obtained from all subjects prior to the experiment.

### 2.2 Behavioral measurements

To assess participants’ daily smartphone use, we administered a self-report questionnaire and supplemented it with a 14-day digital usage log. Participants were instructed to record the total number of hours spent using their smartphones each day during the past two weeks. The questionnaire was completed prior to the fNIRS assessment period. To improve accuracy, participants with compatible smartphones were encouraged to provide usage reports directly from their device settings (e.g., iOS Screen Time, Android Digital Wellbeing) (28, 29). The inclusion of system-generated reports reduces recall bias and provides a more objective estimate of smartphone use compared with self-reports. Based on the reported average daily screen time over the 14-day monitoring period, participants were divided into two groups: High Screen Time Group (HSTG): participants with average screen time > 9 hours per day. Low Screen Time Group (LSTG): participants with average screen time ≤ 9 hours per day. The 9-hour cutoff for daily smartphone use was chosen based on prior evidence (30) showing that individuals with 9 or more hours of daily use demonstrated the highest smartphone addiction scores compared with lower-use group. This threshold was therefore used to define the high-use group for between-group comparisons in WM performance and hemodynamic responses measured via fNIRS.

### 2.3 fNIRS protocol

**Figure 1:**
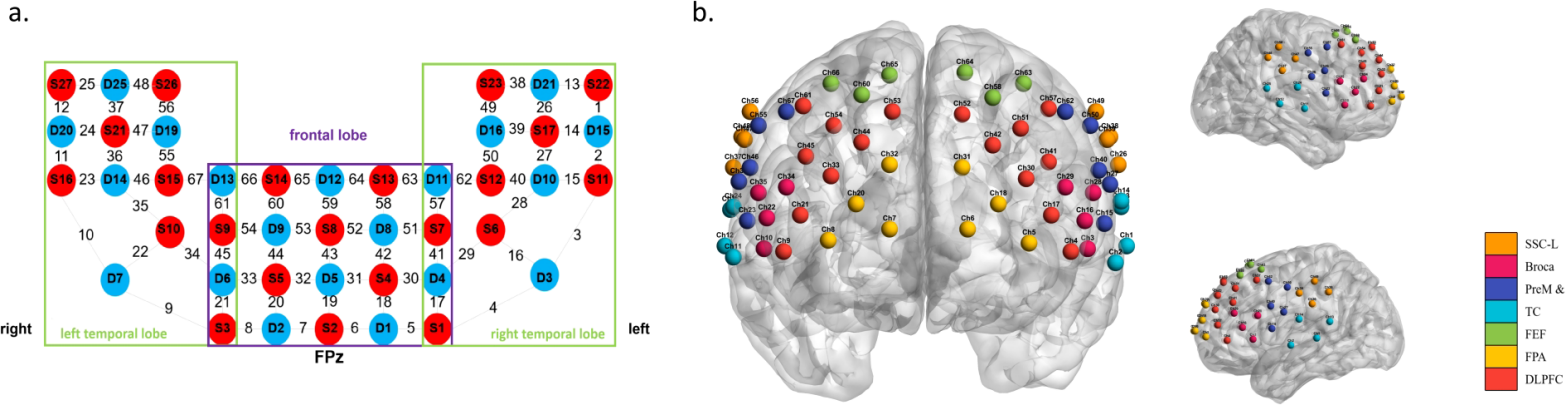
FNIRS channel placement and ROI mapping. **(a)**. Schematic of fNIRS channel arrangement. Red circles represent sources, and blue circles represent detectors. Each source–detector pair forms a measurement channel. The green box outlines the region corresponding to the temporal lobe, and the purple box outlines the region corresponding to the frontal lobe. **(b)**. Schematic representation of ROI distribution. Each circle represents a single fNIRS channel. Different colors indicate distinct regions of interest (ROIs): orange for SSC (somatosensory cortex), pink for Broca, indigo for PreM & SMC (premotor and supplementary motor cortex), cyan for TC (temporal cortex), green for FEF (frontal eye field), yellow for FPA (frontal pole area), and red for DLPFC (dorsolateral prefrontal cortex).

A high-channel fNIRS system (BS-2000, Wuhan Znion Technology Co., Ltd., Wuhan, China) was used to monitor hemodynamic activity during the completion of the 2-back task at two wavelengths (690 and 830 nm). The probe consisted of 27 sources and 25 detectors with 3 cm source-detector-distance, forming 67 measurement channels (see **Figure 1a**). The covered cortical areas included the prefrontal cortex, as well as bilateral temporal and parietal lobes. The 67 channels were then mapped onto the estimated Montreal Neurological Institute (MNI) space (31) using NIRS-SPM (32). Using the Brodmann probabilistic atlas, we grouped these channels into the following seven cortical regions(see **Figure 1b**): dorsolateral prefrontal cortex (DLPFC), frontal pole area (FPA), frontal eye field (FEF), temporal cortex (TC), premotor and supplementary motor cortex (PreM & SMC), primary motor cortex (PMC), Broca area (Broca), and somatosensory cortex (SSC). The system sampled data at 10 Hz. The corresponding Brodmann areas and channel-to-ROI mappings are detailed in **Supplementary Table S1**.

**Figure 2:**
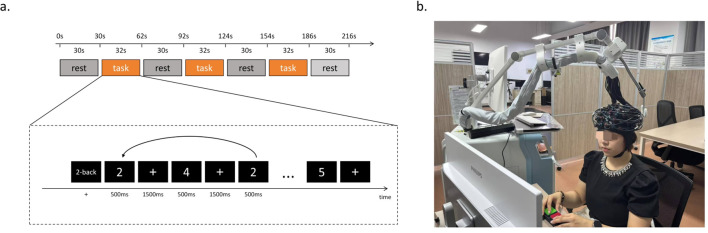
2-back task procedure. **(a)**. Schematic of the 2-back task procedure. The session comprised three 32-second task blocks and four 30-second baseline (rest) blocks, presented alternately. Each task block contains 15 trials. **(b)**. Experimental setup showing a participant wearing the fNIRS device during the 2-back task.

As illustrated in **Figure 2**, WM was evaluated using the 2-back task. The experiment was conducted in a quiet room with consistent lighting to minimize external factors affecting fNIRS measurements. Participants were seated comfortably and instructed to limit head movements and avoid behaviors such as jaw clenching, excessive blinking, or leg movements to reduce motion artifacts. As illustrated in the diagram below, the task session consisted of four 30-second baseline resting blocks interleaved with three 32-second 2-back task blocks. Each task block contained 15 trials. During the resting blocks, participants focused on the “+” fixation cross on the screen. During the task blocks, a sequence of numerical stimuli was presented. For each stimulus, participants were required to decide whether the current number matched the one presented two trials earlier. Each number was displayed for 500 ms, followed by a 1500 ms presentation of the “+” fixation cross, which also served as the inter-stimulus interval. Behavioral performance during the 2-back task was assessed using four parameters: reaction time, accuracy, hit rate, and false alarm rate. Reaction time was defined as the average response latency for correct trials. Accuracy was calculated as the percentage of correct responses across all trials. Hit rate reflected the proportion of correctly identified target stimuli, and false alarm rate represented the proportion of incorrect responses to non-target stimuli.

### 2.4 fNIRS data analysis

#### 2.4.1 Pre-processing and quality control

In this study, MATLAB was used to analyze the fNIRS data. The data were preprocessed using the Homer2 toolbox, including the following steps: (1) The function *hmrIntensity2OD* realized the conservation of light intensity to optical intensity; (2) The function *hmrMotionArtifactByChannel* and *hmrMotionCorrectSpline* were used to detect motion artifacts; Within 0.5 seconds time-window (tMotion), the signal exceeding a threshold in change of 20 standard deviations (STDEVthresh) were marked as motion artifacts. (3) The function *hmrBandpassFilt* was used to remove physiological noise with the frequencies of 0.01–0.1HZ. (4) The function *hmrOD2Conc* helped convert optical intensity to concentrations of oxy-Hb, deoxy-Hb and total Hb based on modified Beer-Lambert law. (5) The function *hmrBlockAvg* was used to average 3 blocks.

Quality control was performed at both the channel and participant levels. At the channel level, a channel was defined as “bad” if excessive motion artifacts were present, operationalized as more than 70% of time points being flagged by the motion detection algorithm. At the participant level, subjects were considered for exclusion if more than 30% of their channels were classified as bad. In the present dataset, channel-level artifact rates were consistently low (mean: 3.63%–21.61%), and the proportion of bad channels per participant ranged from 0–2.99%, well below the 30% exclusion threshold. As no channel or participant met the predefined criteria for exclusion, all 42 subjects were retained for analysis.

#### 2.4.2 Activation analysis

Task-related cortical activation was analyzed using the General Linear Model (GLM) module implemented in NIRS-KIT, a MATLAB-based toolbox for fNIRS data analysis (33). The GLM was computed separately for each channel and participant. Beta coefficients ( β-values) were estimated for each ROI, which reflects the amplitude of the task-evoked hemodynamic response (34). Only HbO signals were analyzed in this study, given their higher sensitivity to cognitive activation compared to HbR (35). To obtain ROI level activation, β-values from all channels belonging to the same ROI were averaged within each participant, which followed the method in previous studies (36).

#### 2.4.3 Functional connectivity analysis

To investigate task-related functional connectivity (FC) patterns, we constructed task-state brain networks using 67 fNIRS channels during the 2-back task. For each participant, a 67 × 67 Pearson correlation matrix was computed by calculating the pairwise correlation coefficients between the time series of each channel pair. For constructing sparsified functional connectivity networks, we referred to the benchmarking study by Luppi & Stamatakis (37), who systematically compared multiple network-construction pipelines using an information-theoretic measure of network representativeness. They reported that an absolute correlation threshold of r = 0.3 produced networks with relatively stable and generalizable topologies. Based on former studies (38, 39), we adopted r= 0.3 as our primary threshold for sparsification, while also performing sensitivity analyses with alternative thresholds (r = 0.25, 0.35) to ensure robustness. Mean functional connectivity values were then computed at different levels. The whole-brain functional connectivity (WBFC) was defined as the average of all pairwise connections across the entire network. To examine hemispheric differences, left-hemisphere functional connectivity (LHFC) and right-hemisphere functional connectivity (RHFC) were calculated as the mean connectivity of all intra-hemispheric edges within the left and right hemispheres, respectively. In addition, inter-hemispheric functional connectivity (InterConn) was defined as the average connectivity strength of all edges linking regions between the two hemispheres. LH-RH Diff was calculated as the difference between the mean functional connectivity of the LHFC and RHFC. All correlation values were Fisher z-transformed to improve normality for statistical analysis.

Based on the resulting weighted connectivity matrices, we extracted several graph-theoretical network metrics to quantify the topological properties of the brain networks (40, 41). These included Global Efficiency ( Eg), which reflects the efficiency of parallel information transfer across the network (42) and defined as:


$$\text{E}g=\frac{1}{N\left(N-1\right)}\underset{i\neq j}{\sum }\frac{1}{L_{i\text{j}}}$$

where $\;L_{ij}$ is the shortest path length between nodes i and j.

Local Efficiency ( Eloc) measures the fault tolerance or resilience of local subnetworks and can be expressed as:


$$Eloc=\frac{1}{N}\underset{i\in G}{\sum }E_{glob}\left(G_{i}\right)$$

where $G_{i}\;$ is the subgraph composed of the neighbors of node i.

The Clustering Coefficient ( Cp) indicates the degree of local interconnectedness among nodes and is calculated as:


$$Cp=\frac{1}{N}\sum_{i=1}^{N}\frac{2e_{i}}{k_{i}\left(k_{i}-1\right)}$$

where $e_{i}$ is the number of edges among the neighbors of node i and $k_{i}$ is the degree of node.

Shortest Path Length ( Lp) represents the average shortest distance between all pairs of nodes, defined as:


$$Lp=\frac{1}{N\left(N-1\right)}\sum_{i\neq j}L_{ij}$$

Finally, Network Density ( D) is the ratio of actual connections to all possible connections and is given by:


$$D=\frac{2E}{N\left(N-1\right)}$$

All metrics were calculated using standard graph analysis functions implemented in the “igraph” R packages (43).

### 2.5 Statistics analysis

All statistical analyses were performed in R (version 4.4.3). To assess task-related activation, two-tailed one-sample t-tests against zero (H_0_: mean β = 0) were performed for each ROI. Between-group differences were examined using two-sample t-tests. Data normality was assessed using the Shapiro-Wilk test. If normality was violated, the Kruskal-Wallis rank sum test was applied. To examine the relationship between daily screen time and neural activation during the 2-back task, Pearson correlation analyses were performed between average daily screen time and activation values extracted from predefined ROIs. Multiple comparisons were corrected using the false discovery rate (FDR) method, with statistical significance set at p< 0.05.

## 3 Result

### 3.1 Demographics information

After excluding six participants who had not enabled the screen time tracking function on their smartphones, the final valid sample consisted of 42 college students, with 18 assigned to the high screen time group (HSTG) and 24 to the low screen time group (LSTG). As shown in **Table 1**, the two groups did not significantly differ in age (t = 0.307, p = 0.761) or gender distribution (χ^2^ = 0.083, p = 0.77). Participants in the HSTG group reported significantly longer daily screen time compared with those in the LSTG group (t= 9.059, p< 0.001).

**Table 1:** Demographics information.

Variable	HSTG	LSTG	Statistic	p
Age (years)	20.000 ± 0.970	20.083 ± 0.717	0.307	0.761
Gender (M/F)	6/12	7/17	0.083	0.77
Screen Time (hour)	11.203 ± 1.379	6.960 ± 1.514	9.0594	0.000

### 3.2 Behavior results

**Figure 3:**
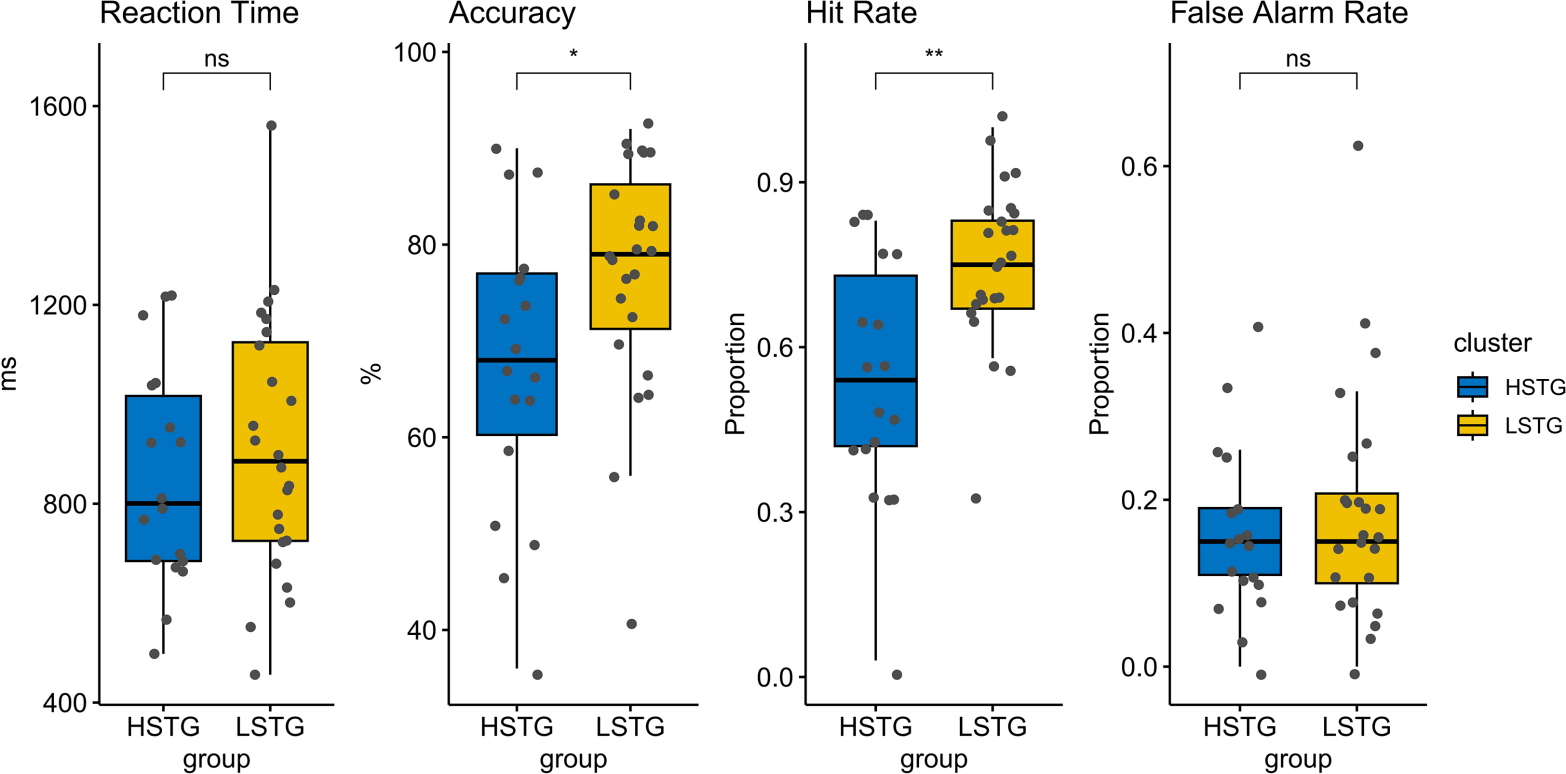
Group differences in behavioral performance on the 2-back task. Boxplots depict the distribution of behavioral performance measures (Reaction Time, Accuracy, Hit Rate, and False Alarm Rate) in the HSTG and LSTG during the 2-back WM task. Group comparisons were performed using independent-samples t-tests. Asterisks indicate levels of statistical significance (*p <.05, **p<.01, ***p <.001).

Behavioral performance during the 2-back task was compared between HSTG and LSTG groups (as shown in **Table 2**, **Figure 3**). The LSTG group showed significantly higher accuracy than the HSTG group (t = -2.24, p = 0.032). Similarly, the Hit Rate was significantly greater in the LSTG group compared to the HSTG group (t = -3.52, p = 0.0015). No significant differences were found between groups for Reaction Time (t = -0.80, p = 0.428) or False Alarm Rate (t = -0.73, p = 0.469). The findings indicate that participants with lower STG connectivity demonstrated better task performance, particularly in terms of accuracy and hit detection, without differences in speed or false alarm rate.

**Table 2:** Group differences in behavioral performance.

Variable	HSTG	LSTG	Statistic	p
Reaction Time (ms)	852.000 ± 221.236	911.750 ± 261.575	-0.801	0.428
Accuracy (%)	67.389 ± 14.888	77.083 ± 12.332	-2.245	0.032*
Hit Rate	0.543 ± 0.219	0.753 ± 0.148	-3.517	0.001**
False Alarm Rate	0.159 ± 0.103	0.186 ± 0.140	-0.731	0.469

HSTG, High Screen Time Group; LSTG, Low Screen Time Group; Asterisks indicate levels of statistical significance (*p<.05, **p<.01, ***p<.001).

### 3.3 fNIRS results

#### 3.3.1 Activation results

To investigate whether the brain activation in specific ROIs significantly differed from zero during the 2-back task, one-sample t-tests of β value were conducted separately for the LSTG and HSTG groups. Several ROIs exhibited significantly above-zero activation in the LSTG group. As mentioned in **Table 3**, the most robust effects were observed in the DLPFC-R (t = 7.59, p_FDR_<0.001), FEF-R (t = 5.59, p_FDR_< 0.001), and SSC-L (t = 5.34, p_FDR_< 0.001). Additional significant activations were found in the PreM & SMC-R, DLPFC-L, FPA-L, FPA-R, SSC-L, SSC-R, Broca-R, and PreM & SMC-L (all p_FDR_< 0.05). Marginal significance was also observed in FEF-L (p_FDR_ =0.013). In contrast, for the HSTG group, only the SSC-L showed a statistically significant positive activation (t = 2.77, p = 0.013), but this effect did not survive correction for multiple comparisons (p_FDR_ = 0.182). These findings suggest that the LSTG group exhibits more widespread and consistent activation across frontal and sensorimotor areas during the 2-back task, whereas the HSTG group shows limited regional engagement.

**Table 4:** Group differences in functional connectivity strength.

Variable	HSTG	LSTG	t	p
WBFC	0.193 ± 0.087	0.260 ± 0.115	-2.047	0.047*
LHFC	0.199 ± 0.090	0.262 ± 0.111	-2.030	0.049*
RHFC	0.213 ± 0.09	0.29 ± 0.118	-2.381	0.022*
InterConn	0.180 ± 0.088	0.251 ± 0.120	-2.210	0.033*
LH-RH Diff	-0.014 ± 0.055	-0.028 ± 0.064	0.737	0.465

WBFC, Whole-brain Functional Connectivity; LHFC, center-hemisphere Functional Connectivity; RHFC, Right-hemisphere Functional Connectivity; InterConn, Inter-hemispheric Functional Connectivity; LH-RH Diff, LH-RH Connectivity Difference. Asterisks indicate statistical significance (*p<.05).

**Figure 4:**
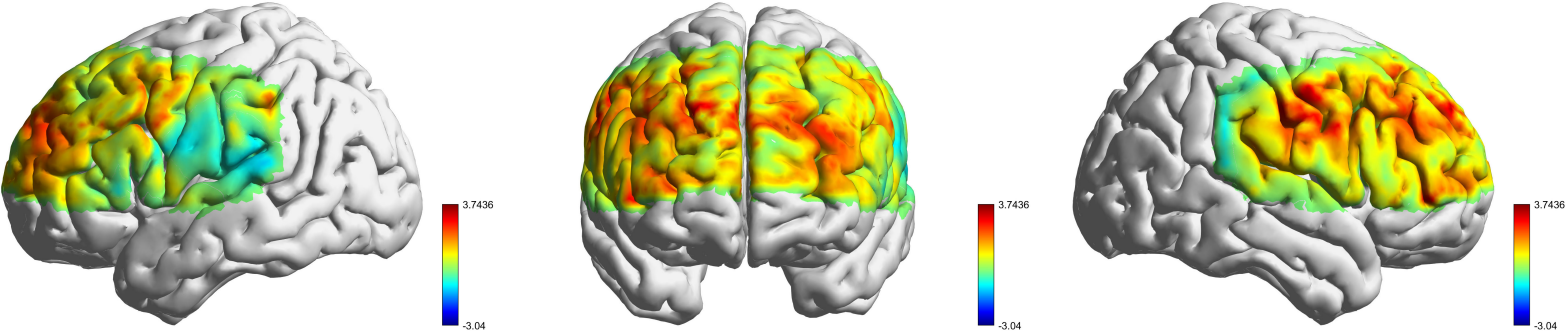
Group comparison of LSTG and HSTG (t-statistics). The panels show the t-statistics from the group comparison (LSTG − HSTG) in three views: left lateral, anterior and right lateral. The color scale indicates t values, with warmer colors (red) reflecting positive values (LSTG > HSTG) and cooler colors (blue) indicating negative values (HSTG > LSTG).

To examine group differences in task-related brain activation during the 2-back task, independent-samples t-tests of β value were conducted comparing the HSTG and LSTG groups across multiple brain areas (as shown in **Figure 4**). Significant differences were observed in both the right and left DLPFC. Specifically, the DLPFC-R showed significantly greater activation in the LSTG group compared to the HSTG group (t= 5.73, p_FDR_< 0.001). Similarly, the DLPFC-L also showed higher activation in the LSTG group than the HSTG group (t = 3.58, p_FDR_ =0.007). In the PreM & SMC-R, LSTG again showed increased activation compared to HSTG (t = 3.41, p_FDR_ = 0.009). Marginal differences were found in several other regions. In the FPA-L, PreM & SMC-L, and FPA-R, the LSTG group showed higher activation than the HSTG group (p< 0.05), but these effects did not survive correction for multiple comparisons (p_FDR_ >0.05).

#### 3.3.2 Functional connectivity results

As shown in **Table 4**, HSTG and LSTG groups differed in functional connectivity measures. WBFC was higher in LSTG than HSTG (t = -2.047, p = 0.047). LHFC was lower in HSTG than LSTG (t = -2.030, p = 0.049), and right-hemisphere connectivity (RHFC) showed a similar reduction (t = -2.381, p = 0.022). InterConn was also reduced in HSTG (t = -2.210, p = 0.033). No significant difference was observed for the left-right hemisphere connectivity difference (t = 0.737, p = 0.465). Sensitivity analyses using alternative thresholds (r = 0.25 and r = 0.35) largely confirmed these results (see **Supplementary Tables S2**, **S3**). Except for LHFC at r = 0.25, which lost statistical significance, the patterns of group differences in WBFC, RHFC, and InterConn were consistent with those observed at r = 0.3, indicating the robustness of the functional connectivity findings across thresholds.

**Table 5:** Group differences in graph-theoretical metrics.

Variable	HSTG	LSTG	t	p
Global efficiency	1.544 ± 0.115	1.593 ± 0.100	-1.461	0.153
Local Efficiency	1.706 ± 0.072	1.698 ± 0.077	0.336	0.739
Clustering Coefficient	0.655 ± 0.098	0.723 ± 0.108	-2.124	0.040*
Shortest Path Length	0.728 ± 0.068	0.691 ± 0.057	1.825	0.077
Network Density	0.365± 0.135	0.467 ± 0.169	-2.184	0.035*

Group differences were assessed using independent-sample t-tests. Asterisks indicate statistical significance (*p<.05).

#### 3.3.3 Brain network characteristics

We compared the graph-theoretical metrics between the HSTG and LSTG groups. Consistent with the values reported in **Table 5**, Global Efficiency and Local Efficiency did not differ significantly between groups. In contrast, HSTG showed a significantly lower clustering coefficient (t = -2.124, p = 0.040) and network density (t = -2.184, p = 0.035) than LSTG, indicating a less segregated and sparser network organization. Shortest Path Length also showed a marginal trend toward being higher in HSTG. Alternative thresholds (r = 0.25 and r = 0.35) confirmed these findings. Both clustering coefficient and network density remained significantly lower in HSTG compared to LSTG at these thresholds, and the pattern of shortest path length differences showed a similar trend, indicating the robustness of the graph-theoretical results across thresholds.

**Table 3:** Group differences in task-evoked activation (β-values).

ROI	HSTG	LSTG	t	p	p_FDR_
DLPFC-R	-0.003 ± 0.026	0.046 ± 0.03	5.734	0.000***	0.000***
DLPFC-L	0.008 ± 0.045	0.073 ± 0.072	3.584	0.001***	0.007**
PreM & SMC-R	-0.013 ± 0.05	0.034 ± 0.033	3.406	0.002**	0.009**
FPA-L	0.006 ± 0.062	0.05 ± 0.051	2.467	0.019*	0.056
PreM & SMC-L	-0.004 ± 0.054	0.041 ± 0.066	2.422	0.020*	0.056
FPA-R	-0.008 ± 0.066	0.035 ± 0.045	2.364	0.025*	0.059
FEF-R	0.018 ± 0.058	0.047 ± 0.041	1.805	0.081	0.163
FEF-L	0.001 ± 0.117	0.058 ± 0.102	1.669	0.104	0.183
TC-L	0.09 ± 0.215	0.002 ± 0.133	1.538	0.136	0.205
SSC-L	0.025 ± 0.039	0.044 ± 0.04	1.483	0.146	0.205
Broca-R	-0.001 ± 0.102	0.034 ± 0.049	1.351	0.190	0.242
TC-R	0.008 ± 0.074	0.054 ± 0.276	0.782	0.441	0.514
Broca-L	0.003 ± 0.074	-0.019 ± 0.231	0.440	0.663	0.714
SSC-R	0.025 ± 0.109	0.023 ± 0.027	0.078	0.939	0.939

ROI, Region of Interest; BA.ID, Brodmann Area; T, t-value from two-sample t-test; p, uncorrected p-value; p_FDR_, p-value adjusted using FDR; Asterisks indicate levels of statistical significance (*p<.05, **p<.01, ***p<.001).

### 3.4 Correlation analysis

**Figure 5:**
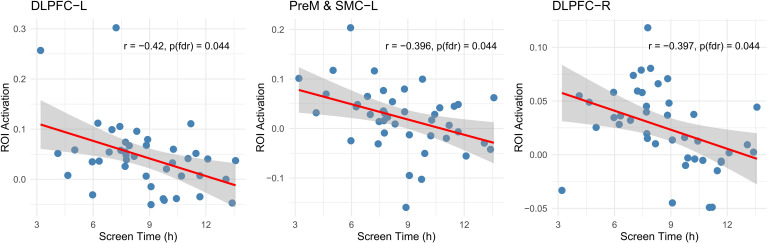
Correlations between daily screen time and ROI activations. Scatter plots illustrate the associations between average daily screen time and ROI activation. Significant negative correlations were observed in the left DLPFC, left PreM/SMC, and right DLPFC.

As shown in **Figure 5**, higher daily screen time was significantly associated with reduced activation in the left DLPFC (r = -0.42, p_FDR_ = 0.044), left PreM/SMC (r = -0.40, p_FDR_ = 0.044), and right DLPFC (r = -0.40, p_FDR_ = 0.044). A negative trend was also observed in the right PreM/SMC (r = -0.35, p_FDR_ = 0.083). These findings suggest that increased screen exposure may be linked to lower engagement of prefrontal and premotor regions during working-memory processing.

## 4 Discussion

The present study examined the impact of screen time on working memory (WM) and its neural correlates by comparing high and low screen-time groups. Convergent behavioral and neuroimaging findings indicate that excessive screen exposure is associated with reduced WM efficiency and altered brain network organization.

Behaviorally, LSTG participants demonstrated higher accuracy and hit rate during the 2-back task, which are in line with prior findings (16, 17). Although some earlier studies have reported mixed associations (8, 9) between screen use and WM, differences in study design likely contribute to these discrepancies. Prior longitudinal or modality-specific studies examined different forms of media (8), developmental windows, or WM tasks (9, 44, 45), whereas the present study examined screen time over the past two weeks, capturing short-term effects more directly. Given evidence for a dose–response relationship (46) between screen exposure and executive function, the threshold used in this study may have further highlighted performance differences.

Two theoretical pathways may account for the observed behavioral impairments. The cognitive resource depletion or “brain drain” hypothesis proposes that the mere presence or frequent use of digital devices consumes limited executive resources, thereby weakening the ability to maintain goal-relevant information (6, 47). Empirical work shows that even resisting phone-checking can diminish performance on unrelated tasks, particularly among individuals with higher dependence on smartphones (17). Additionally, the scan-and-shift hypothesis suggests that fast-paced digital environments promote habitual attentional shifting, increasing susceptibility to bottom-up attentional capture at the expense of sustained, top-down control (6, 48). Such attentional styles may undermine performance in WM tasks that require inhibition and continuous monitoring (49).

At the neural activation level, participants with low screen time exhibited significantly greater activation in bilateral DLPFC and right PreM & SMC regions, which are essential for WM maintenance, manipulation, and the coordination of sequential actions (50–54) The reduced activation observed in high screen-time users likely reflects insufficient recruitment of frontoparietal-motor systems needed for demanding executive control. Although some previous work, such as Guo et al. (24), interpreted reduced DLPFC activation as evidence of increased neural efficiency, converging findings from recent developmental and naturalistic studies challenge this interpretation. Research in preschoolers suggests that higher screen exposure is associated with poorer inhibitory control and reduced prefrontal activation (6). Similarly, naturalistic fNIRS studies in young adults show immediate declines in n-back and Go/No-Go performance following social media use, accompanied by reduced activation in dlPFC, vlPFC, and IFG (55). These studies support the view that intensive digital engagement may transiently or cumulatively disrupt prefrontal recruitment rather than enhance efficiency (7, 55–57).

Functional connectivity findings reinforce this perspective. Low screen-time participants exhibited higher whole-brain, intra-hemispheric, and inter-hemispheric connectivity, whereas high screen-time users showed reduced network integration across large-scale neural systems. Weaker connectivity suggests diminished coordination between regions supporting executive control, hindering the ability to flexibly allocate cognitive resources during WM tasks. While Guo et al. (24) reported increased connectivity between specific frontal regions in individuals with pathological smartphone use, this pattern was interpreted as a compensatory or advantageous effect. In contrast, broader neuroimaging evidence indicates that problematic smartphone or social media use is associated with reduced connectivity within and between key executive networks, including salience, central executive, and dorsal attention systems (54, 58). Converging fNIRS and fMRI findings (54, 58–60) therefore suggest that diminished connectivity reflects reduced network engagement rather than functional optimization.

Graph-theoretical analyses provided further insights into large-scale network organization. Although global and local efficiency did not differ significantly between groups, high screen-time users displayed lower clustering coefficient, reduced density, and a trend toward longer characteristic path length. In graph theory, reduced clustering suggests weaker local specialization, while longer path lengths indicate reduced efficiency of global information transfer—both of which can compromise the rapid integration of distributed neural signals. Lower network density reflects a sparser architecture, providing fewer communication pathways and potentially limiting cognitive flexibility (61–65). Together, these alterations highlight a disrupted balance between functional segregation and integration in individuals with high screen exposure, consistent with diminished support for complex executive operations.

Although the present study focused on total smartphone screen exposure, recent sedentary behavior theoretical frameworks (66) provide a useful interpretive context for understanding the observed associations. The dual-axis taxonomy of sedentary behavior highlights substantial cognitive heterogeneity across activities, characterized by differences in mental activation and content relevance (e.g., cognitively demanding activities such as reading academic materials or problem solving versus passive, low-relevance activities such as continuous short-video scrolling) (66–69). A similar framework may be informative for understanding screen-based exposure, which can involve both mental activation and content relevance/goal directedness. When only total screen duration is assessed, these qualitatively distinct behaviors are aggregated, which may partially account for the observed reductions in working memory performance and prefrontal engagement in the high screen-time group. From this perspective, the reduced activation and connectivity observed in high screen-time participants may reflect diminished executive engagement associated with a greater proportion of cognitively passive or attentional-fragmenting screen use, rather than an effect of screen exposure duration alone. Importantly, this interpretation remains inferential, as the present study did not directly assess the cognitive characteristics of screen-based activities. To better capture this heterogeneity, future studies could adopt more nuanced and multidimensional measurement to optimize experimental design. These may include app-category-based usage logs to differentiate functional screen activities, purpose-based self-reports assessing goal directedness, and composite indices integrating mental activation and content relevance. Such measures could then be systematically examined in relation to working memory performance and fNIRS-derived activation, functional connectivity, and graph-theoretical network metrics, allowing for a more direct test of how distinct patterns of screen exposure map onto executive functioning and brain network organization.

Beyond describing the cognitive impact, our findings offer significant translational value. Altered patterns of prefrontal activation and network organization identified by fNIRS may serve as neural indicators associated with reduced executive engagement in individuals with higher screen exposure. Importantly, brain-related measures need not be viewed solely as outcomes (67); rather, they may also help identify individuals who could benefit from additional support in optimizing digital habits or cognitive self-regulation. Gaining insight in this direction may inform more targeted, theory-guided approaches to intervention and monitoring, complementing behavioral assessments in educational or high digital-demand settings. Collectively, these findings highlight the potential role of neuroimaging markers in advancing research and practice aimed at promoting cognitive health in the digital age.

Several limitations should be acknowledged. First, as mentioned above, this study assessed only total smartphone screen time and did not differentiate screen-based activities by cognitive engagement or content relevance. Given that screen exposure is a heterogeneous construct, aggregating diverse activities into a single duration-based metric may obscure specific associations with working memory performance and related neural processes (66–68). Future studies should therefore employ more fine-grained measurement approaches. The second limitation of this study is the absence of measurements related to sleep quality and psychological status (e.g., depression or anxiety), which may influence cognitive performance. Future studies should include standardized assessments of sleep and mood to better account for individual variability. Finally, the cross-sectional design prevents causal inference, leaving open the possibility that pre-existing neural or cognitive differences contribute to higher screen use rather than result from it. Future longitudinal studies with larger samples and finer-grained assessments of media type are needed to clarify these relationships.

Conclusion: Within the sample of undergraduate student, high screen exposure correlates with reduced working memory accuracy, weaker prefrontal engagement, and disrupted network organization, suggesting less efficient neural strategies. These effects may stem from the nature of screen interactions-cognitively passive or low-relevance use, making screen use a modifiable factor affecting cognition and brain networks.

## Data Availability

The raw data supporting the conclusions of this article will be made available by the authors, without undue reservation.

## References

[1] Qiu Y Xie YJ Chen L Wang SL Yang H Huang Z . Electronic media device usage and its associations with BMI and obesity in a rapidly developing city in south China. Front Public Health. (2020) 8:551613. doi: 10.3389/fpubh.2020.551613, PMID: 33490008 PMC7820191

[2] China Internet Information Center . The 56th statistical report on internet development in China released. Available online at: https://www.cnnic.net.cn/n4/2025/0721/c88-11328.html (Accessed January 8, 2026).

[3] Kumban W Cetthakrikul S Santiworakul A . Smartphone addiction, screen time, and physical activity of different academic majors and study levels in university students. Int J Environ Res Public Health. (2025) 22:237. doi: 10.3390/ijerph22020237, PMID: 40003463 PMC11855490

[4] Yuan Y He X He Q Jia Y Xu Z Li M . Problematic mobile phone use and time management disposition in Chinese college students: the chain mediating role of sleep quality and cognitive flexibility. BMC Psychol. (2023) 11:440. doi: 10.1186/s40359-023-01481-z, PMID: 38093382 PMC10720238

[5] Dong H Zheng H Wang M Ye S Dong GH . The unbalanced behavioral activation and inhibition system sensitivity in internet gaming disorder: Evidence from resting-state Granger causal connectivity analysis. Prog Neuropsychopharmacol Biol Psychiatry. (2022) 119:110582. doi: 10.1016/j.pnpbp.2022.110582, PMID: 35661790

[6] Meng X Liang X Liu C Cheng N Lu S Zhang K . Associations between screen media use and young children’s inhibitory control: Evidence from behavioral and fNIRS study. Comput Hum Behav. (2024) 152:108041. doi: 10.1016/j.chb.2023.108041

[7] Xiang MQ Lin L Song YT Hu M Hou XH . Reduced left dorsolateral prefrontal activation in problematic smartphone users during the Stroop task: An fNIRS study. Front Psychiatry. (2023) 13:1097375. doi: 10.3389/fpsyt.2022.1097375, PMID: 36699489 PMC9868828

[8] Sarvajna DH Winston JS S DP Nuza M Venugopalan V . Screen time exposure and domain-specific working memory in young adults. Cureus. (2024) 16:e60626. doi: 10.7759/cureus.60626, PMID: 38903378 PMC11187442

[9] Soares PSM De Oliveira PD Wehrmeister FC Menezes AMB Gonçalves H . Screen time and working memory in adolescents: A longitudinal study. J Psychiatr Res. (2021) 137:266–72. doi: 10.1016/j.jpsychires.2021.02.066, PMID: 33725639

[10] Wang H Sun Y Lan F Liu Y . Altered brain network topology related to working memory in internet addiction. J Behav Addict. (2020) 9:325–38. doi: 10.1556/2006.2020.00020, PMID: 32644933 PMC8939409

[11] Yeung MK Han YMY . Changes in task performance and frontal cortex activation within and over sessions during the n-back task. Sci Rep. (2023) 13:1–14. doi: 10.1038/s41598-023-30552-9, PMID: 36849731 PMC9971214

[12] Baddeley A . Working memory: theories, models, and controversies. Annu Rev Psychol. (2012) 63:1–29. doi: 10.1146/annurev-psych-120710-100422, PMID: 21961947

[13] Friedman NP Robbins TW . The role of prefrontal cortex in cognitive control and executive function. Neuropsychopharmacology. (2022) 47:72–89. doi: 10.1038/s41386-021-01132-0, PMID: 34408280 PMC8617292

[14] Blume F Irmer A Dirk J Schmiedek F . Day-to-day variation in students’ academic success: The role of self-regulation, working memory, and achievement goals. Dev Sci. (2022) 25:e13301. doi: 10.1111/desc.13301, PMID: 35780513

[15] Sharifian N Zahodne LB . Social media bytes: daily associations between social media use and everyday memory failures across the adult life span. J Gerontol B Psychol Sci Soc Sci. (2020) 75:540–8. doi: 10.1093/geronb/gbz005, PMID: 30624708 PMC7021445

[16] Abramson MJ Benke GP Dimitriadis C Inyang IO Sim MR Wolfe RS . Mobile telephone use is associated with changes in cognitive function in young adolescents. Bioelectromagnetics. (2009) 30:678–86. doi: 10.1002/bem.20534, PMID: 19644978

[17] Ward AF Duke K Gneezy A Bos MW . Brain drain: the mere presence of one’s own smartphone reduces available cognitive capacity. J Assoc Consum Res. (2017) 2:140–54. doi: 10.1086/691462

[18] Canale N Vieno A Doro M Rosa Mineo E Marino C Billieux J . Emotion-related impulsivity moderates the cognitive interference effect of smartphone availability on working memory. Sci Rep. (2019) 9:18519. doi: 10.1038/s41598-019-54911-7, PMID: 31811205 PMC6898282

[19] Pablo JN Shires J Castellanos J Kapila M Kemmelmeier LL Berryhill ME . Changed detection: No relationship between working memory and media usage in Covid-era and contemporary young adults. Atten Percept Psychophys. (2025) 87:1098–106. doi: 10.3758/s13414-025-03063-0, PMID: 40169525

[20] Ball K Berch DB Helmers KF Jobe JB Leveck MD Marsiske M . Effects of cognitive training interventions with older adults: a randomized controlled trial. JAMA. (2002) 288:2271–81. doi: 10.1001/jama.288.18.2271, PMID: 12425704 PMC2916176

[21] Rebok GW Ball K Guey LT Jones RN Kim HY King JW . Ten-year effects of the advanced cognitive training for independent and vital elderly cognitive training trial on cognition and everyday functioning in older adults. J Am Geriatr Soc. (2014) 62:16–24. doi: 10.1111/jgs.12607, PMID: 24417410 PMC4055506

[22] Boot WR Kramer AF Simons DJ Fabiani M Gratton G . The effects of video game playing on attention, memory, and executive control. Acta Psychol (Amst). (2008) 129:387–98. doi: 10.1016/j.actpsy.2008.09.005, PMID: 18929349

[23] Green CS Bavelier D . Effect of action video games on the spatial distribution of visuospatial attention. J Exp Psychol Hum Percept Perform. (2006) 32:1465–78. doi: 10.1037/0096-1523.32.6.1465, PMID: 17154785 PMC2896828

[24] Guo W Zhang W Zhang J Li Z Zhu W . Effective connectivity analysis of verbal working memory advantage across materials for pathological smartphone users by fNIRS. Psychiatry Res: Neuroimaging. (2023) 336:111731. doi: 10.1016/j.pscychresns.2023.111731, PMID: 37875058

[25] Li Y Li S Yuan Z Zhao S Wan F Xu T . IVCAN: An improved visual curve attention network for fNIRS-Based motor imagery/execution classification. Biomed Signal Process Control. (2025) 104:107679. doi: 10.1016/j.bspc.2025.107679

[26] Li Y Sun Y Wan F Yuan Z Jung TP Wang H . MetaNIRS: A general decoding framework for fNIRS based motor execution/imagery. Neural Networks. (2025) 192:107873. doi: 10.1016/j.neunet.2025.107873, PMID: 40683191

[27] S Z Q W C K A G Y S R Z . Age-related cerebral changes during different n-back tasks: a functional near-infrared spectroscopy study. Front Aging Neurosci. (2024) 16:1437587. doi: 10.3389/fnagi.2024.1437587, PMID: 39478697 PMC11521811

[28] Ohme J Araujo T de VCH Piotrowski JT . Mobile data donations: Assessing self-report accuracy and sample biases with the iOS Screen Time function. SAGE J. (2020) 9:293–313. doi: 10.1177/2050157920959106

[29] Walsh LC Okabe-Miyamoto K Regan A Twenge J Lyubomirsky S . The association between well-being and objectively measured versus self-reported smartphone time. PsyArXiv. (2021). doi: 10.31234/osf.io/zcwmv

[30] Kaya S Kaya M . Investigation of smartphone addiction levels among university students. IJPES. (2020) 7:14–25. doi: 10.17220/ijpes.2020.03.002

[31] Ak S M O H D V J I D . Spatial registration of multichannel multi-subject fNIRS data to MNI space without MRI. NeuroImage. (2005) 27:842–851. doi: 10.1016/j.neuroimage.2005.05.019, PMID: 15979346

[32] Jc Y S T Ke J J J J J . NIRS-SPM: statistical parametric mapping for near-infrared spectroscopy. NeuroImage. (2009) 44:428–447. doi: 10.1016/j.neuroimage.2008.08.036, PMID: 18848897

[33] Hou X Zhang Z Zhao C Duan L Gong Y Li Z . NIRS-KIT: a MATLAB toolbox for both resting-state and task fNIRS data analysis. Neurophotonics. (2021) 8:010802. doi: 10.1117/1.NPh.8.1.010802, PMID: 33506071 PMC7829673

[34] Von Lühmann A Ortega-Martinez A Boas DA Yücel MA . Using the general linear model to improve performance in fNIRS single trial analysis and classification: A perspective. Front Hum Neurosci. (2020) 14:30. doi: 10.3389/fnhum.2020.00030, PMID: 32132909 PMC7040364

[35] Nakahachi T Ishii R Iwase M Canuet L Takahashi H Kurimoto R . Frontal cortex activation associated with speeded processing of visuospatial working memory revealed by multichannel near-infrared spectroscopy during Advanced Trail Making Test performance. Behav Brain Res. (2010) 215:21–7. doi: 10.1016/j.bbr.2010.06.016, PMID: 20600348

[36] Hou S Chen S Huang Z Yin X Zhao K Zou J . Mapping the neural mechanisms of creativity by convergent and divergent thinking in school-aged children: A functional near-infrared spectroscopy study. Think Skills Creativ. (2023) 49:101300. doi: 10.1016/j.tsc.2023.101300

[37] Luppi AI Stamatakis EA . Combining network topology and information theory to construct representative brain networks. Netw Neurosci. (2021) 5:96–124. doi: 10.1162/netn_a_00170, PMID: 33688608 PMC7935031

[38] Cohen AD Yang B Fernandez B Banerjee S Wang Y . Improved resting state functional connectivity sensitivity and reproducibility using a multiband multi-echo acquisition. NeuroImage. (2021) 225:117461. doi: 10.1016/j.neuroimage.2020.117461, PMID: 33069864 PMC10015256

[39] Penalba-Sánchez L Oliveira-Silva P Sumich AL Cifre I . Increased functional connectivity patterns in mild Alzheimer’s disease: A rsfMRI study. Front Aging Neurosci. (2023) 14:1037347. doi: 10.3389/fnagi.2022.1037347, PMID: 36698861 PMC9869068

[40] Ismail LE Karwowski W . A graph theory-based modeling of functional brain connectivity based on EEG: A systematic review in the context of neuroergonomics. IEEE Access. (2020) 8:155103–35. doi: 10.1109/ACCESS.2020.3018995

[41] Rubinov M Sporns O . Complex network measures of brain connectivity: Uses and interpretations. NeuroImage. (2010) 52:1059–69. doi: 10.1016/j.neuroimage.2009.10.003, PMID: 19819337

[42] Xu T Huang J Pei Z Chen J Li J Bezerianos A . The effect of multiple factors on working memory capacities: aging, task difficulty, and training. IEEE Trans BioMed Eng. (2023) 70:1967–78. doi: 10.1109/TBME.2022.3232849, PMID: 37015624

[43] Csárdi G Nepusz T Traag V Horvát S Zanini F Noom D . igraph: network analysis and visualization (2022). Available online at: https://igraph.org (Accessed January 8, 2026).

[44] Scharinger C Soutschek A Schubert T Gerjets P . Comparison of the working memory load in N-back and working memory span tasks by means of EEG frequency band power and P300 amplitude. Front Hum Neurosci. (2017) 11:6. doi: 10.3389/fnhum.2017.00006, PMID: 28179880 PMC5263141

[45] Redick TS Lindsey DRB . Complex span and n-back measures of working memory: A meta-analysis. Psychon Bull Rev. (2013) 20:1102–13. doi: 10.3758/s13423-013-0453-9, PMID: 23733330

[46] Law EC Han MX Lai Z Lim S Ong ZY Ng V . Associations between infant screen use, electroencephalography markers, and cognitive outcomes. (2023) 177:311–8. doi: 10.1001/jamapediatrics.2022.5674, PMID: 36716016 PMC9887532

[47] Wang X Zhao X Yu C . The influence of information and social overload on academic performance: The role of social media fatigue, cognitive depletion, and self-control. Rev Psicodidáctica (Engl ed). (2025) 30:500164. doi: 10.1016/j.psicoe.2025.500164

[48] Kwon M Jung YC Lee D Lee J . Altered resting-state functional connectivity of the dorsal anterior cingulate cortex with intrinsic brain networks in male problematic smartphone users. Front Psychiatry. (2022) 13:1008557. doi: 10.3389/fpsyt.2022.1008557, PMID: 36262635 PMC9573940

[49] Beyens I Valkenburg PM Piotrowski JT . Screen media use and ADHD-related behaviors: Four decades of research. Proc Natl Acad Sci USA. (2018) 115:9875–81. doi: 10.1073/pnas.1611611114, PMID: 30275318 PMC6176582

[50] Owen AM McMillan KM Laird AR Bullmore E . N-back working memory paradigm: a meta-analysis of normative functional neuroimaging studies. Hum Brain Mapp. (2005) 25:46–59. doi: 10.1002/hbm.20131, PMID: 15846822 PMC6871745

[51] Hardwick RM Rottschy C Miall RC Eickhoff SB . A quantitative meta-analysis and review of motor learning in the human brain. Neuroimage. (2013) 67:283–97. doi: 10.1016/j.neuroimage.2012.11.020, PMID: 23194819 PMC3555187

[52] Cañas A Juncadella M Lau R Gabarrós A Hernández M . Working memory deficits after lesions involving the supplementary motor area. Front Psychol. (2018) 9:765. doi: 10.3389/fpsyg.2018.00765, PMID: 29875717 PMC5974158

[53] Nakajima R Okita H Kinoshita M Miyashita K Nakada M Yahata T . Direct evidence for the causal role of the left supplementary motor area in working memory: A preliminary study. Clin Neurol Neurosurg. (2014) 126:201–4. doi: 10.1016/j.clineuro.2014.09.009, PMID: 25306857

[54] Ahn J Lee D Namkoong K Jung YC . Altered functional connectivity of the salience network in problematic smartphone users. Front Psychiatry. (2021) 12:636730. doi: 10.3389/fpsyt.2021.636730, PMID: 34349676 PMC8326368

[55] Aitken A Jounghani AR Carbonell LM Kumar A Crawford S Bowden AK . The effect of social media consumption on emotion and executive functioning in college students: an fNIRS study in natural environment. Res Sq. (2024). doi: 10.21203/rs.3.rs-5604862/v1, PMID: 39764144 PMC11703342

[56] Ding K Shen Y Gao J Li H . Impact of digital addiction tendency on inhibitory control in preschoolers: An fNIRS study. Brain Res. (2025) 1865:149868. doi: 10.1016/j.brainres.2025.149868, PMID: 40825500

[57] Li H Wu D Yang J Luo J Xie S Chang C . Tablet use affects preschoolers’ Executive function: fNIRS evidence from the dimensional change card sort task. Brain Sci. (2021) 11:567. doi: 10.3390/brainsci11050567, PMID: 33946675 PMC8146550

[58] Lee D Lee J Namkoong K Jung YC . Altered functional connectivity of the dorsal attention network among problematic social network users. Addictive Behav. (2021) 116:106823. doi: 10.1016/j.addbeh.2021.106823, PMID: 33460991

[59] Li X Li Y Wang X Hu W . Reduced brain activity and functional connectivity during creative idea generation in individuals with smartphone addiction. Soc Cognit Affect Neurosci. (2023) 18:nsac052. doi: 10.1093/scan/nsac052, PMID: 36149062 PMC9619470

[60] Chun JW Choi J Cho H Choi MR Ahn KJ Choi JS . Role of frontostriatal connectivity in adolescents with excessive smartphone use. Front Psychiatry. (2018) 9:437. doi: 10.3389/fpsyt.2018.00437, PMID: 30258373 PMC6143708

[61] J Z L L L Q Y K B L D Y . The topological organization of white matter network in internet gaming disorder individuals. Brain Imaging Behav. (2017) 11:1769–78. doi: 10.1007/s11682-016-9652-0, PMID: 27815774

[62] Li Y Shang Y Yang Y . Clustering coefficients of large networks. Inf Sci. (2017) 382–383:350–8. doi: 10.1016/j.ins.2016.12.027

[63] Watts DJ Strogatz SH . Collective dynamics of ‘small-world’ networks. Nature. (1998) 393:440–2. doi: 10.1038/30918, PMID: 9623998

[64] Sporns O Zwi JD . The small world of the cerebral cortex. Neuroinform. (2004) 2:145–62. doi: 10.1385/NI:2:2:145, PMID: 15319512

[65] Farrar DC Mian AZ Budson AE Moss MB Koo BB Killiany RJ . Retained executive abilities in mild cognitive impairment are associated with increased white matter network connectivity. Eur Radiol. (2018) 28:340–7. doi: 10.1007/s00330-017-4951-4, PMID: 28695358 PMC5798454

[66] Zhang Z Chen Y Yu Q Li J Zou L Mavilidi MF . A neurobiological taxonomy of sedentary behavior for brain health. Trends Neurosci. (2025) 48:853–64. doi: 10.1016/j.tins.2025.09.002, PMID: 41038753

[67] Zou L Herold F Cheval B Wheeler MJ Pindus DM Erickson KI . Sedentary behavior and lifespan brain health. Trends Cognit Sci. (2024) 28:369–82. doi: 10.1016/j.tics.2024.02.003, PMID: 38431428 PMC11778811

[68] Hallgren M Dunstan DW Owen N . Passive versus mentally active sedentary behaviors and depression. Exercise Spt Sci Rev. (2020) 48:20–7. doi: 10.1249/JES.0000000000000211, PMID: 31663866

[69] Wang J Herold F Zhang Z Chen Y Pindus DM Hillman CH . Prospective associations between screen-based sedentary behaviors and cognitive performance among children aged 5–7 years. Ment Health Phys Activ. (2025) 29:100686. doi: 10.1016/j.mhpa.2025.100686

