# Supplementary material for: The impact of smartphone use on working memory in college students: a functional near-infrared spectroscopy study
Source: Front Psychiatry. 2026 Jan 26;16:1725048. doi: 10.3389/fpsyt.2025.1725048 (PMC12883783; doi:10.3389/fpsyt.2025.1725048)
Supplement: Supplementary file 1 [file Table1.docx]

**Table S1: Channel Coordinates and Zone Index**

| **ChID** | **MNI_x** | **MNI_y** | **MNI_z** | **ROI** | **BA** |  | **ChID** | **MNI_x** | **MNI_y** | **MNI_z** | **ROI** | **BA** |
| --- | --- | --- | --- | --- | --- | --- | --- | --- | --- | --- | --- | --- |
| 1 | -71 | -30 | 3 | TC-L | 21 |  | 35 | 61 | 22 | 22 | Broca-R | 44_45 |
| 2 | -67 | -8 | -3 | TC-L | 21 |  | 36 | 68 | -2 | 26 | PreM & SMC-R | 6 |
| 3 | -57 | 24 | 2 | Broca-L | 44_45 |  | 37 | 70 | -27 | 31 | SSC-R | 1 |
| 4 | -51 | 46 | 1 | DLPFC-L | 46 |  | 38 | -65 | -40 | 42 | SSC-L | 1 |
| 5 | -36 | 64 | 4 | FPA-L | 10 |  | 39 | -64 | -15 | 41 | SSC-L | 1 |
| 6 | -14 | 73 | 9 | FPA-L | 10 |  | 40 | -61 | 9 | 30 | PreM & SMC-L | 6 |
| 7 | 14 | 74 | 9 | FPA-R | 10 |  | 41 | -43 | 41 | 33 | DLPFC-L | 46 |
| 8 | 36 | 66 | 5 | FPA-R | 10 |  | 42 | -23 | 54 | 39 | DLPFC-L | 46 |
| 9 | 52 | 48 | 1 | DLPFC-R | 46 |  | 43 | 0 | 56 | 40 | DLPFC | 46 |
| 10 | 59 | 26 | 2 | Broca-R | 44_45 |  | 44 | 24 | 55 | 40 | DLPFC-R | 46 |
| 11 | 70 | -8 | -1 | TC-R | 21 |  | 45 | 44 | 41 | 35 | DLPFC-R | 46 |
| 12 | 73 | -29 | 3 | TC-R | 21 |  | 46 | 64 | 9 | 31 | PreM & SMC-R | 6 |
| 13 | -69 | -41 | 17 | TC-L | 21 |  | 47 | 66 | -16 | 41 | SSC-R | 1 |
| 14 | -69 | -15 | 19 | TC-L | 21 |  | 48 | 67 | -39 | 42 | SSC-R | 1 |
| 15 | -63 | 8 | 11 | PreM & SMC-L | 6 |  | 49 | -60 | -28 | 51 | SSC-L | 1 |
| 16 | -56 | 33 | 12 | Broca-L | 44_45 |  | 50 | -58 | -5 | 46 | PreM & SMC-L | 6 |
| 17 | -44 | 54 | 14 | DLPFC-L | 46 |  | 51 | -33 | 39 | 45 | DLPFC-L | 46 |
| 18 | -25 | 67 | 18 | FPA-L | 10 |  | 52 | -12 | 49 | 50 | DLPFC-L | 46 |
| 19 | 1 | 66 | 20 | FPA | 10 |  | 53 | 13 | 49 | 51 | DLPFC-R | 46 |
| 20 | 26 | 68 | 18 | FPA-R | 10 |  | 54 | 34 | 40 | 46 | DLPFC-R | 46 |
| 21 | 46 | 55 | 14 | DLPFC-R | 46 |  | 55 | 61 | -5 | 46 | PreM & SMC-R | 6 |
| 22 | 58 | 35 | 13 | Broca-R | 44_45 |  | 56 | 64 | -30 | 51 | SSC-R | 1 |
| 23 | 65 | 10 | 12 | PreM & SMC-R | 6 |  | 57 | -43 | 21 | 52 | DLPFC-L | 46 |
| 24 | 70 | -14 | 17 | TC-R | 21 |  | 58 | -23 | 35 | 56 | FEF-L | 8 |
| 25 | 71 | -40 | 16 | TC-R | 21 |  | 59 | 1 | 39 | 55 | FEF | 8 |
| 26 | -68 | -26 | 32 | SSC-L | 1 |  | 60 | 24 | 35 | 57 | FEF-R | 8 |
| 27 | -65 | -2 | 25 | PreM & SMC-L | 6 |  | 61 | 45 | 23 | 53 | DLPFC-R | 46 |
| 28 | -59 | 20 | 22 | Broca-L | 44_45 |  | 62 | -49 | 11 | 51 | PreM & SMC-L | 6 |
| 29 | -49 | 40 | 24 | Broca-L | 44_45 |  | 63 | -34 | 17 | 61 | FEF-L | 8 |
| 30 | -35 | 56 | 27 | DLPFC-L | 46 |  | 64 | -13 | 26 | 65 | FEF-L | 8 |
| 31 | -12 | 64 | 31 | FPA-L | 10 |  | 65 | 14 | 28 | 64 | FEF-R | 8 |
| 32 | 14 | 65 | 32 | FPA-R | 10 |  | 66 | 35 | 18 | 61 | FEF-R | 8 |
| 33 | 35 | 57 | 28 | DLPFC-R | 46 |  | 67 | 51 | 11 | 51 | PreM & SMC-R | 6 |
| 34 | 51 | 42 | 24 | Broca-R | 44_45 |  |  |  |  |  |  |  |
