# Supplementary material for: The impact of smartphone use on working memory in college students: a functional near-infrared spectroscopy study
Source: Front Psychiatry. 2026 Jan 26;16:1725048. doi: 10.3389/fpsyt.2025.1725048 (PMC12883783; doi:10.3389/fpsyt.2025.1725048)
Supplement: Supplementary file 2 [file Table2.docx]

**Table S2: Summary of functional connectivity strength and** **graph-theoretical metrics(r=0.25)**

| variable | HSTG | LSTG | t | p |
| --- | --- | --- | --- | --- |
| **functional connectivity strength** | | | | |
| WBFC | 0.208±0.086 | 0.274±0.114 | -2.041 | 0.048* |
| LHFC | 0.214±0.09 | 0.275±0.11 | -1.986 | 0.054 |
| RHFC | 0.228±0.087 | 0.303±0.116 | -2.386 | 0.022* |
| InterConn | 0.194±0.088 | 0.265±0.12 | -2.201 | 0.034* |
| LH-RH Diff | -0.014±0.053 | -0.028±0.064 | 0.755 | 0.455 |
| **graph-theoretical metrics** | | | | |
| Global efficiency | 1.841±0.09 | 1.861±0.085 | -0.707 | 0.484 |
| Local Efficiency | 1.95±0.077 | 1.919±0.075 | 1.333 | 0.191 |
| Clustering Coefficient | 0.674±0.094 | 0.743±0.108 | -2.189 | 0.035* |
| Shortest Path Length | 0.599±0.035 | 0.588±0.033 | 0.974 | 0.337 |
| Network Density | 0.418±0.134 | 0.518±0.166 | -2.156 | 0.037* |
