# Supplementary material for: The impact of smartphone use on working memory in college students: a functional near-infrared spectroscopy study
Source: Front Psychiatry. 2026 Jan 26;16:1725048. doi: 10.3389/fpsyt.2025.1725048 (PMC12883783; doi:10.3389/fpsyt.2025.1725048)
Supplement: Supplementary file 3 [file Table3.docx]

**Table S3: Summary of functional connectivity strength and graph-theoretical metrics(r=0.35)**

| variable | HSTG | LSTG | t | p |
| --- | --- | --- | --- | --- |
| **functional connectivity strength** | | | | |
| WBFC | 0.177±0.086 | 0.243±0.115 | -2.03 | 0.049* |
| LHFC | 0.182±0.088 | 0.246±0.11 | -2.072 | 0.045* |
| RHFC | 0.197±0.091 | 0.273±0.119 | -2.337 | 0.025* |
| InterConn | 0.164±0.088 | 0.234±0.12 | -2.179 | 0.035* |
| LH-RH Diff | -0.015±0.054 | -0.027±0.064 | 0.681 | 0.5 |
| **graph-theoretical metrics** | | | | |
| Global efficiency | 1.315±0.132 | 1.374±0.12 | -1.496 | 0.144 |
| Local Efficiency | 1.518±0.088 | 1.52±0.086 | -0.092 | 0.927 |
| Clustering Coefficient | 0.637±0.099 | 0.705±0.11 | -2.082 | 0.044* |
| Shortest Path Length | 0.872±0.104 | 0.812±0.104 | 1.835 | 0.075 |
| Network Density | 0.315±0.133 | 0.416±0.169 | -2.16 | 0.037* |
